# Revised Centrality Measures Tell a Robust Story of Ion Conduction in Solids

**DOI:** 10.1021/acs.jpcb.3c03886

**Published:** 2023-10-19

**Authors:** Konrad Sebastian Gomez-Haibach, Maria Alexandra Gomez

**Affiliations:** † Department of Mathematics, Worcester Polytechnic Institute, Worcester, Massachusetts 01609, United States; ‡ Department of Chemistry, Mount Holyoke College, South Hadley, Massachusetts 01075, United States

## Abstract

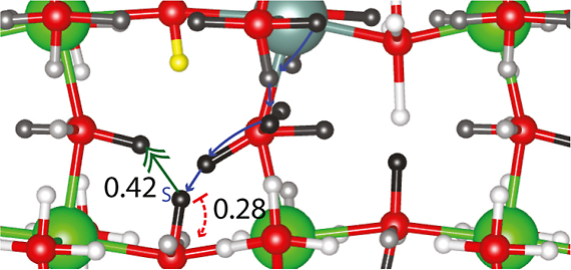

The three most commonly used centrality measures in network theory have been adapted to consider ion conduction time rather than the number of steps. Flow-IN centrality highlights sites with the largest flow of ions from the nearest neighbor sites. Return-flow centrality highlights sites with a fast rate of first returns for the conducting ion. Flow-through centrality highlights which sites support significant flow of conducting ions and appears more robust to removal of the most central vertices. Exploring these centrality measures with the sample system of proton conduction in yttrium doped barium zirconate shows flow-through centrality to provide a robust picture with high contrast between sites involved in the most probable long-range periodic conduction paths and kinetic Monte Carlo trajectories versus sites rarely visited. The flow-through centrality, including all paths further highlights that when the most central proton site is filled, the remaining highest flow-through centrality sites are nearby, corroborating earlier studies suggesting proton pair motion. Finally, while both return-flow and flow-through centrality measure images deteriorate with noise, image restoration is possible when a detailed balance is used to calculate the smaller rate constant in a forward/backward pair.

## Introduction

Molecular dynamics (MD) simulations have been used to probe ion conduction in many systems, including ionic solids.^1−5^ In systems where moves can be cataloged, kinetic Monte Carlo (KMC) allows different time scale moves by choosing a move based on its probability and advancing the simulation clock based on the move’s rate constant.^6−9^ To overcome the challenges of both the distinct time and length scale important to ion conduction, a variety of multiscale methods have been developed and applied successfully to shed light on critical ion conduction technologies.^10,11^ Many of these methods generate trajectories that can be probed to understand the system dynamics. Trajectories of ion conduction in solids may include many conducting ion diffusion steps near traps and a few escapes to fast conduction regions intermingled with smaller movements of other ions. One time-based centrality measure has been shown to highlight traps and conduction highways in a single picture, giving an overview of the ion conduction landscape.^12,13^ However, network theory has developed many centrality measures based on number of steps to highlight different network features.^14−17^ This paper shows how the three most common types of step-based centrality measures can be converted to time-based centrality measures to probe significant features of ion conduction in solids and their sensitivity to noisy data. Taken as viewpoints of a whole, these measures provide a more robust story of ion conduction, both for single-ion motion in an ionic solid and for ion–ion correlated motion. Long-range path searches and KMC are used to corroborate the story.

**Figure 1:**
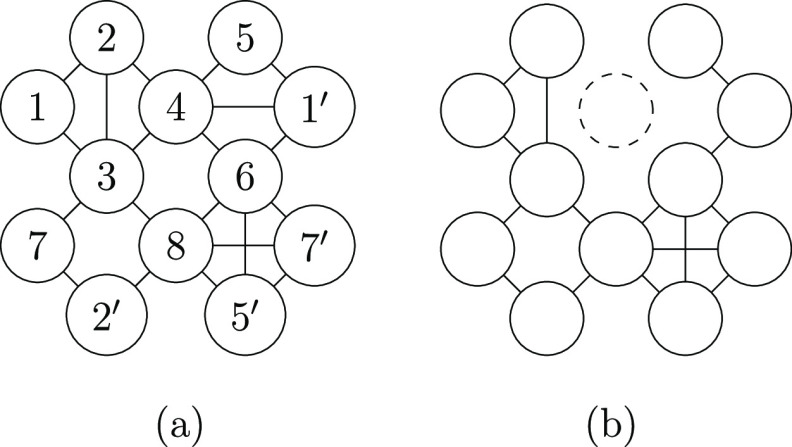
Removing one vertex from the ergodic graph (a) yields the graph (b), which features one inaccessible site.

Ion conduction through a material can be represented as movement through a graph, where each vertex corresponds to the minimum energy structure, with the conducting ion on a specific binding site. An edge connects two vertices if the minima can be connected via a single transition state. Site probabilities indicate the likelihood of finding the system in the particular minimum or binding site structure, while the rate constant for transition between sites (*k*_*i*,*j*_) characterizes the rate of motion between sites.^12,13,18−21^Figure 1a shows a sample conduction graph for an ergodic system, where all vertices are available and accessible from any other vertex. To characterize large systems without unusual edges, these graphs need to include the same periodic boundary conditions common to large system simulations.^1^ The periodic graph in Figure 1a represents the simulation cell of a periodic system with 8 unique vertices or binding sites. The 1′, 7′, 2′, and 5′ sites are periodic images of vertices 1, 7, 2, and 5. The images are obtained when the simulation cell graph is shifted and replicated to the right and bottom of the original. A single ion moving in an ionic solid is said to conduct long-range when its conduction path starts on a unique vertex of the periodic graph and ends on any periodic image of that vertex, while the path spans the full simulation box. Periodic pathways can be linked together to create long-range conduction pathways, which are characteristic of real system conduction if the simulation cell is sufficiently large.^19^ Real physical system graphs are much larger and KMC motion through the graph exhibits significant random diffusion through sites, as seen in our earlier work.^12,13^ To consider conducting an ion–ion correlation or how one conduction ion influences the pathway of another, the edges to the occupied vertex are effectively removed from the graph, as seen in Figure 1b. Both scenarios depicted in Figure 1 will be considered.

The three most common types of centrality measures used in networks are degree, closeness, and betweenness.^14−17^ The degree of a vertex is determined by the number of connections to the site. For directed graphs, the degree-IN is the number of connections leading into the vertex in a single step, and the degree-OUT is the number of connections leading out from the vertex. Closeness centrality weighs each site based on its “closeness” to all other sites, which is thought to be a measure of how fast information travels from a single site. More specifically, closeness centrality is the inverse of the average number of steps a vertex takes from all other vertices in the graph. The network theory betweenness centrality of a site *i* is the fraction of paths which pass through the site *i* and can be calculated over different sets of paths. Sites with high betweenness centrality allow for a significant flow of information. In information networks, these sites are frequently targeted as their removal would likely disrupt information passage.

Because reactions or moves of an ion from one site to another are not identical but depend on the specific move rate constant, our work translates the three common centrality measures, based on number of steps, to related measures based on time and applies them in the common simulation periodic boundary condition context. Because the centrality measures are now based on time, we also change their names to give intuition for what each is measuring. Degree-IN and degree-OUT are changed to adjacent flow-IN and adjacent flow-OUT. Adjacent flow-IN for site *i* is defined as the rate of flow of adjacent sites to site *i* and adjacent flow-OUT as the rate of flow from *i* to adjacent sites. An adjacent site is directly connected to the vertex of interest. Closeness centrality is the inverse of the average number of steps a vertex is from all other vertices in the graph and is similar to the inverse of the average time to first return to a vertex, the subject of our earlier work.^12^ The latter measure, however, considers both flow away and flow back to the vertex. Intuitively, this centrality measure indicates how easily an ion starting at this vertex can flow to other vertices and return to the original vertex (or its periodic image). Hence, we refer to this as return-flow centrality, which considers flow to and from any other site.

The final common centrality measure, betweenness centrality, is meant to characterize how important a site is to the flow of information and will be called flow-through centrality. Flow-through centrality will be calculated with two path sets: (1) all possible periodic paths of length *N*, which traverse the full simulation box without interior cycles (simplified long-range conduction paths) and (2) all possible paths. Our earlier work^13,19^ has used a dynamic programming approach for finding the set (1) and sorting paths by probability. Flow-through centrality for site *i* for these long-range periodic paths is defined here as the sum over the probabilities of all of the paths going through site *i*. The resulting sum is effectively the probability of paths going through *i*. To define flow-through centrality for site *t* over all paths [set (2)], we consider the ratio of the mean time to travel between any sites *i* and *j* through *t* to the mean time to travel between *i* and *j*. This ratio indicates whether flow through *t* increases or decreases the conduction time. The ratio is averaged through all possible sites *i* and *j* and the inverse of this average increase or decrease in conduction time is the flow-through centrality for site *t* for all paths. Hence, the flow-through centrality is high when there is a decrease in the conduction time and low when there is an increase in the conduction time. This flow-through centrality highlights how much a vertex aids or hinders the flow of ions.

In summary, we will compare how well a vertex allows ions to flow out and return (return-flow centrality) with how well a vertex aids the flow of ions through the vertex (flow-through centrality) and with local flow into and out of a vertex from or to immediately adjacent vertices (adjacent flow-IN and flow-OUT centrality). Flow-through centrality will be calculated both over long-range conduction pathways and over all pathways. These centrality measures will be used to access different aspects of conductivity for a proton in yttrium-doped barium zirconate with and without an excess proton. The excess proton effectively removes the graph vertex. The robustness of return-flow and flow-through centrality through all paths was measured by adding random noise to the rate constants used. Finally, a detailed balance is used to recover the centrality measures in the presence of moderate noise scenarios.

## Methods

### Overview of System and Data Used to Calculate Centrality Measures

Adjacent flow-IN, adjacent flow-OUT, return-flow, and flow-through centrality measures will be found to characterize the flow of a single proton in a 12.5% yttrium-doped barium zirconate, as well as the flow of an excess proton in the same system with an initial proton fixed at the most probable site for the single proton. The purpose of this work is to access the type of information which can be obtained from each of these time centrality measures as well as the robustness of the most useful to noise. To calculate these centrality measures, the energies of structures with a proton at each of the possible binding sites as well as the transition states is needed. Earlier work found ab initio Perdew–Burke–Ernzerhof density functional site energies and transition-state energies between sites for a single proton^18^ and an excess proton^21^ in 12.5% yttrium-doped barium zirconate under very similar conditions. While other studies have calculated energies differently, for the purposes of this study, which evaluates centrality measures as a way to understand conduction, using a consistent set of energies is most useful. With binding energies *E*_*i*_ and transition-state energy (*E*_*ij*_^TS^) between sites *i* and *j*, the rate constant *k*_*ij*_ for motion between sites *i* and *j* can be found using a harmonic transition-state theory^6^ as  where *k*_B_ is the Boltzmann’s constant and *T* is the temperature.  and  are the harmonic vibration frequencies at the minimum and the transition state, respectively. It is common practice to save computational work and approximate the full vibrational frequency ratio based on a typical range.^6^ A previous work suggests that the frequencies are on a scale of 1.0 ps^–1^. Because the prefactor has one more frequency in the numerator compared to the denominator, an estimate of 1.0 ps^–1^ is used.^13^ Because the probability of moving between sites is proportional to the rate constant between those sites, we define the normalized probability to move from *i* to *j* as *p*_*ij*_ = *k*_*ij*_/∑_*n*_*k*_*in*_. Normalization ensures that the probability of moving to any site from *i* is 1. The probability to start at any site *i* is given by the Boltzmann distribution for a harmonic system or .^6,12^ For ease of calculation, we continue to use a frequency scale of 1 ps^–1^ and hence, *Q* is the normalization factor, ensuring that the sum over all site probabilities is 1. A typical conduction temperature of 1000 K is used. With these quantities, each type of centrality is calculated as described in the next method sections. To obtain a centrality image for each type of centrality, the smallest centrality is subtracted from all, and the result is divided by the highest centrality. The resulting scaled centrality value is used to set the grayscale for each site, with 0 being white or least central and 1 being black or most central. Finally, to access how robust return-flow and flow-through centrality measures are to noise, these are calculated with and without Gaussian noise with zero mean and 0.02, 0.10, and 0.20 ps^–1^ standard deviation in the rate constants. A detailed balance (π_*i*_*k*_*ij*_ = π_*j*_*k*_*ji*_) is used to mitigate the noise by calculating the smaller rate constant from the larger one, which in principle is less susceptible to noise using the equation *k*_*ij*_^small^ = π_*j*_*k*_*ji*_^large^/π_*i*_.

### Adjacent Flow-IN and Flow-OUT Centrality Measures Highlight Sites with the Greatest Nearest Neighbor Inward and Outward Ion Flow

Adjacent flow-IN centrality for site *i* is the sum of the rate constants from neighboring sites to site *i*, while adjacent flow-OUT centrality for site *i* is the sum of the rate constants from *i* to the neighboring sites, as seen in eq 1.
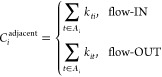
1*A*_*i*_ is the set of sites adjacent to *i*. Adjacency to *i* means that there is a single one-step connection or a single transition state to *i* from the starting point. Adjacent flow-IN and flow-OUT centrality measures give us the overall rate of flow from or to adjacent sites to or from site *i*, respectively. While this is the simplest centrality measure to calculate, the measure only considers the flow from or to adjacent sites, which may not represent how central the site is to the full system. These two centrality measures are the only nearest neighbor measures.

### Return-Flow Centrality Highlights Sites with the Fastest First Returns

Return-flow centrality of *i* is the inverse of the average time to first returns to *i*. The average time to first return to *i* is the average of the mean time to first return to *i* after visiting any other site *j* for the first time or . The mean time to first return to *i* after visiting site *j* for the first time is the sum of the mean time to go from *i* to *j* for the first time (*m*_*ij*_) and the meantime to go from *j* to *i* for the first time (*m*_*ji*_). The return flow centrality is given in these terms in eq 2.
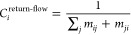
2

In the appendix of our earlier work,^12^ we showed that
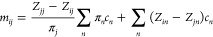
3where *Z* = (*I* – *P* + *W*)^−1^. *Z* is the fundamental matrix for the ergodic chains. *I* is the identity matrix. *P* is the matrix of probabilities of going from site *i* to site *j*. *W* is a matrix whose rows are π, the vector of the site probabilities. *c*_*n*_ is the expected time for a first step starting at *n* or , where *k*_*nl*_ is the rate constant for motion from site *n* to site *l*. The inverse of the rate constant for a specific move is the average time of that move. Note that  as in our earlier work.^12^ The most central site for return-flow centrality is the vertex with the shortest average time of the first returns. Both trapped ions and ions on periodic highways return to the sites quickly. At trapped sites, ions never go far from the initial site, while on periodic highways, ions are moving at high speeds and thus return to the original sites through periodic boundary conditions quickly. Hence, return-flow centrality highlights both traps and long-range conduction highways, as seen in previous works.^12,13^

### Flow-through Centrality Highlights Sites through which a Set of Paths Flows Most

Flow-through centrality quantifies the flow of paths through sites and is calculated for two path sets: (1) periodic long-range paths^19^ of length *N* that span the simulation box and (2) all paths. We define flow-through centrality for site *i* for the first path set as the sum of probabilities for each of the paths containing site *t*, as shown in eq 4.
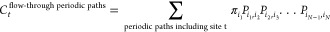
4where *i*_1_, *i*_2_, ...*i*_*N*_ are the first through *N* vertices or sites in the paths, π_*i*_ is the probability of having an ion at site *i*, and *P*_*i*,*j*_ is the probability of an ion moving from site *i* to site *j*. The dynamic programming scheme described in our earlier work^13,19^ is used to find these periodic long-range paths. The method entails a search for connections to successive sites in the path while removing previously visited sites from the search to avoid interior loops. The final site in the *N* step pathway must periodically connect to the first site to ensure that the path can be periodically replicated, creating a long-range pathway through many simulation boxes.

Finding all possible paths of the system would be a more challenging task, and so to calculate flow-through centrality over the set of all paths (set 2), we make use of *m*_*ij*_, the mean time to go from *i* to *j* for the first time, as shown in eq 3. The flow-through centrality for a site *t* is the inverse of the average ratio of the mean time to go from *i* to *j* through *t* to the mean time to go from *i* to *j*, as shown in eq 5.
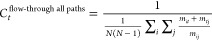
5when the ratio, , flow through site *t* slows the conduction between *i* and *j*. When the ratio, , flow through site *t* accelerates conduction between *i* and *j*. When one of the end points is *t*, the ratio is 1 as *m*_*tt*_ = 0. Paths with the same initial and final end points are excluded. Hence, the centrality or inverse of the average ratio is larger when site *t* increases the flow and smaller when flow is decreased.

## Results and Discussion

### Understanding the Example System

**Figure 2:**
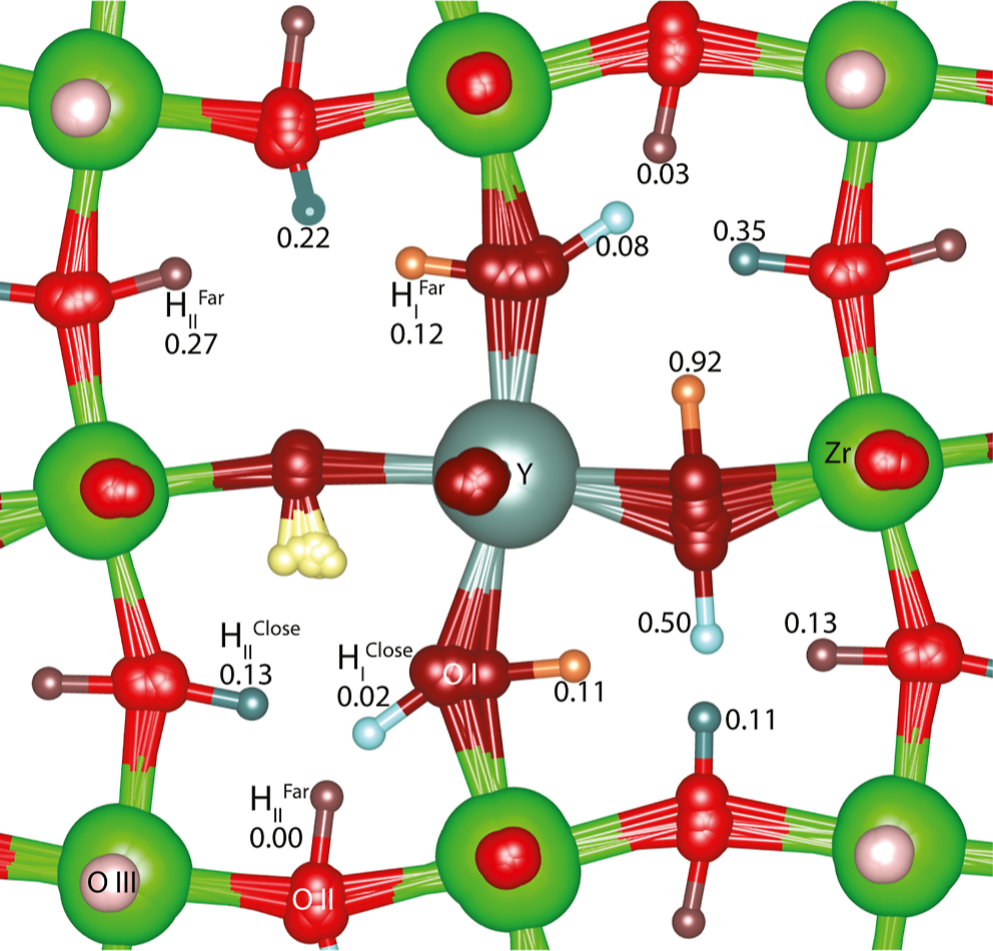
Overlapped images of the minima with one proton fixed at the site highlighted in yellow are shown with the site label and relative energy. Between the sites is the relative transition-state energy. All energies are given in eV.

An overlay plane of 14 minima or vertices of a 12.5% yttrium-doped barium zirconate system with two protons is shown in Figure 2 to give a sense of the ionic solid considered. One of the protons in the 14 minima is fixed at the yellow site, while the second proton site is distinct for each minimum. The yellow site is the lowest energy single proton site.^18^ One ion of each type of proton is shown in Figure 2. The yttrium ion is in the center and in teal. Zirconium ions are in green. Both yttrium and zirconium ions are surrounded by six oxygen ions, forming an octahedron. Distinct oxygen-ion types are labeled I, II, or III based on whether they are the nearest, second nearest, or third nearest to the yttrium-ion dopant. Further, the distinct types of protons are labeled with a subscript based on the type of oxygen ion to which they are bonded and a superscript indicating whether the oxygen ion opposite the hydroxide is close or far.^22^ The H_I_^Far^ proton site occupied in all of the minima is highlighted in yellow. The other H_I_^Far^ sites are orange. Notice that the presence of the fixed yellow proton effectively removes a vertex from the conduction graph, which changes the energy of the otherwise equivalent H_I_^Far^ sites significantly. The range of energies for the orange H_I_^Far^ sites seen in Figure 2 is 0.11 to 0.92 eV. *H*_I_^close^, *H*_II_^Far^, and *H*_II_^Close^ sites are highlighted in blue, brown, and teal, respectively. Sites of each type have different relative energies^21^ due to their distinct position relative to the yellow proton. The relative energies are listed in Figure 2. The relative energies of the sites in the absence of the symmetry-breaking yellow excess proton are 0.00, 0.02, 0.13, 0.20 eV for sites H_I_^Far^, *H*_II_^Far^, *H*_I_^Close^, and *H*_II_^Close^, respectively.^18^

To move from one minimum to an adjacent minimum, a proton can transfer from one oxygen to another on the same octahedron, while the other ions relax to attain a new local minimum. For example, a possible intraoctahedral transfer of a proton in the bottom left quadrant of Figure 2 moves the proton from the *H*_II_^Far^0.00 eV site to the *H*_II_^Close^0.13 eV site while shifting other ions. The move is said to be intraoctahedral transfer (T) because the line defined by the initial and the final proton sites is on an edge of an octahedron around a single zirconium ion. In contrast, a move in the same lower left quadrant from the *H*_II_^Close^0.13 eV site to the *H*_I_^Close^0.02 eV site moves a proton from one octahedral center to another. In this case, the transfer is said to be interoctahedral transfer (I). Only 14 of the 92 minima in the system are overlaid in Figure 2. Absent are proton sites above and below the oxygen ions in the plane, giving each oxygen ion four possible binding sites where only one can be occupied at a time. To move to these sites, the proton would need to rotate around its bonding oxygen ion. Overall, proton conduction occurs through a series of transfers (T and/or I) and rotations (R).

For comparison with centrality measure images, the long-range *N*-step periodic long-range paths and KMC trajectories for a proton starting at the *H*_IID_^Far^ site with and without the yellow fixed proton at the closest H_I_^Far^ site were calculated as in our earlier work.^13,19,21^Tables 1 and 2 show the average estimated times and average limiting barriers for *N* = 8–15 step periodic long-range paths with and without the fixed yellow protons at the closest H_I_^Far^ to the starting *H*_IID_^Far^, respectively. Additionally, these tables show the fraction of the time that the long-range periodic path motion is limited by rotation (R), intraoctahedral transfer (T), and interoctahedral transfer (I) steps. Finally, the tables show the KMC average estimated time to cross the simulation box along with the time average limiting barrier to crossing and the fraction of the time that a crossing is limited by R, T, or I steps. Five KMC trajectories were started at *H*_IID_^Far^ with and without a fixed proton at the closest H_I_^Far^ site. The tables highlight a single standard deviation for the KMC averages. Both tables show that while there are one or two path lengths that give the shortest time averages for periodic long-range paths, the KMC trajectory average time per crossing is closer to the longer time averages for *N*-step periodic long-range paths, highlighting that real-time crossings are not direct passages but do some nonproductive diffusive motion. Hence, the fast periodic long-range paths should only be considered as simplifications of the actual long-range paths. The KMC trajectories like the slower long-range periodic paths show an increase in limiting barrier to long-range motion from 0.34 to 0.41 eV when a proton is fixed at the yellow H_I_^Far^ site when starting from the closest *H*_IID_^Far^ site. Prior work weighing the initial site by its Boltzmann probability, considering all starting points, and including calculated frequencies showed a similar pattern but different averages.^21^ KMC simulations when multiple protons move show a radial distribution function with a broad first neighbor peak between 2 and 10 Å showing a significant range of two proton distances and increasing long-range limiting barriers with increasing number of protons.^21^ A more recent work^23^ further emphasizes proton-pair motion with an increased limiting barrier.

**Table 1:** Average Estimated Times in ps, Average Limiting Barriers in eV, and Fraction of Long-Range Paths with Limiting Barrier of *R*, *T*, and *I* Are Shown for all Paths of N Steps Starting at *H*_IID_^Far^ with a Fixed Proton at the Closest H_I_^Far^ Site^a^

*N*	⟨ET⟩ (ps)	⟨LB⟩ (eV)	*f*_R_	*f*_T_	*f*_I_
8	311	0.42	0.00	1.00	0.00
9	346	0.42	0.00	0.55	0.45
10	**117**	0.31	0.82	0.18	0.00
11	247	0.38	0.06	0.35	0.58
12	162	0.33	0.57	0.43	0.00
13	273	0.39	0.04	0.42	0.54
14	227	0.36	0.25	0.71	0.04
15	305	0.39	0.05	0.44	0.51
KMC	280 (10)	0.41 (0.01)	0.10 (0.04)	0.66 (0.06)	0.23 (0.06)

a The shortest average estimated times are bolded. The last row includes the KMC average estimated time per crossing, time average limiting barrier, and fraction of the time that long-range path motion is limited by *R*, *T*, or *I* steps. Five KMC trajectories were started at *H*_IID_^Far^ with a fixed proton at the closest H_I_^Far^ site. A single standard deviation is given in parentheses.

**Table 2:** Average Estimated Times, Average Limiting Barriers, and Fraction of Long-Range Paths with Limiting Barrier of *R*, *T*, and *I* Are Shown for all Paths of N Steps Starting at a *H*_IID_^Far^^a^

*N*	⟨*ET*⟩ (ps)	⟨LB⟩ (eV)	*f*_R_	*f*_T_	*f*_I_
8	**105**	0.31	0.09	0.90	0.01
9	141	0.33	0.03	0.84	0.13
10	**104**	0.30	0.29	0.70	0.00
11	157	0.32	0.08	0.55	0.37
12	129	0.31	0.21	0.78	0.01
13	167	0.32	0.12	0.53	0.36
14	156	0.32	0.18	0.80	0.02
KMC	150 (20)	0.34 (0.01)	0.04 (0.01)	0.85 (0.04)	0.11 (0.03)

a The shortest average estimated times are bolded. The last row includes the KMC average estimated time per crossing, time average limiting barrier, and fraction of the time that a path motion is limited by *R*, *T*, or *I* steps. Five KMC trajectories were started a *H*_IID_^far^ site. A single standard deviation is given in parentheses.

Next, we compare and contrast adjacent flow, return flow, and flow-through time-based centrality measures for the system of an excess proton^21^ shown in Figure 2 and a system with a single proton, i.e., one where all sites are accessible.^18^ Removing the fixed yellow proton in Figure 2 would give additional 4 sites, giving 96 sites in the single proton graph. All centrality images show the proton binding sites possible in grayscale with black featuring the highest centrality and white the lowest.

### What Adjacent Flow, Return Flow, and Flow-through Centrality Indicate about Ion Conduction

**Figure 3:**
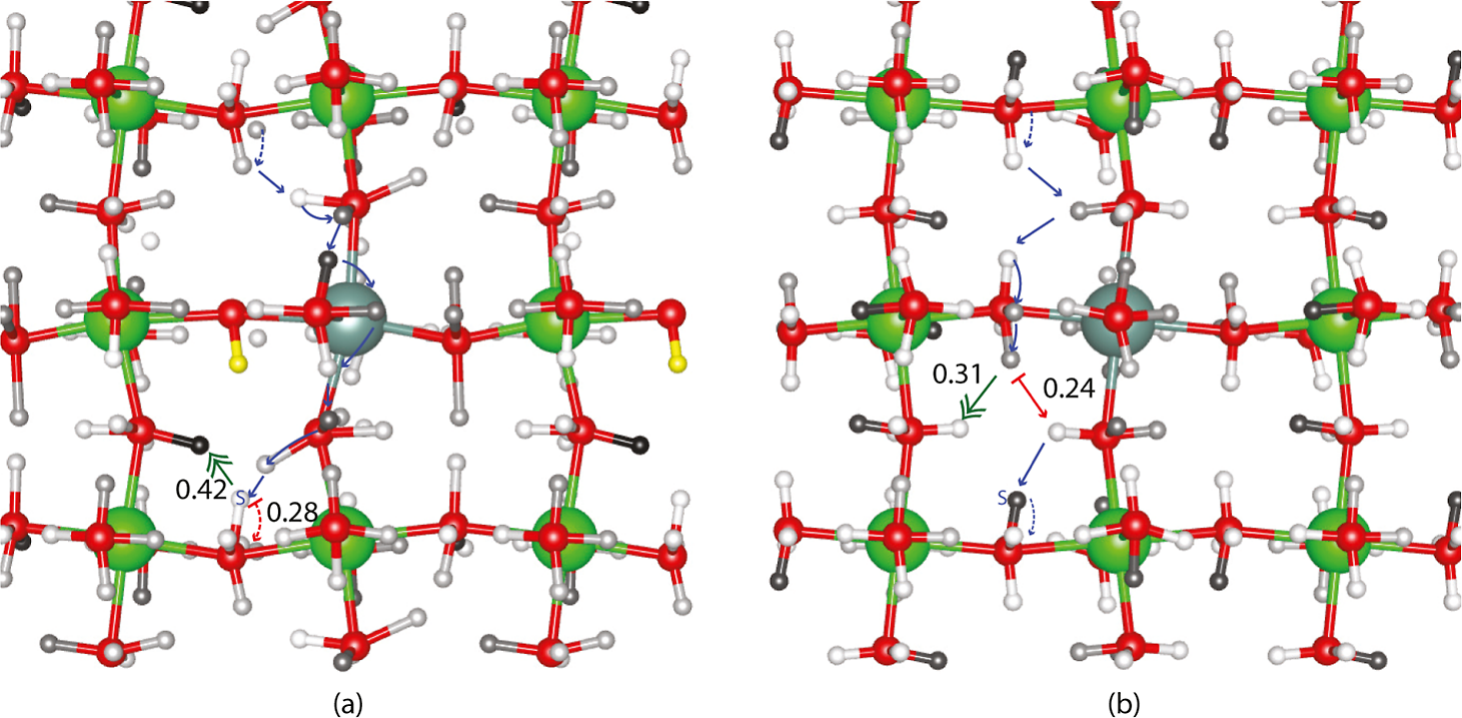
Adjacent flow-IN centrality around a removed vertex (fixed proton) is shown in a dopant plane in (a). Adjacent flow degree-IN centrality with all sites available is shown in (b). Additionally, (a) most probable fastest 10-step periodic path and (b) most probable fastest 8-step periodic path with and without the fixed yellow proton, respectively. For both blue paths, the starting point is noted by an S and the limiting barrier move is signaled in red. The most often cataloged KMC limiting barrier is highlighted with a double-headed green arrow.

Figure 3 shows the adjacent flow-IN centrality for proton sites for the system with a fixed proton (yellow) in (a) and with all proton sites available in (b). As in all of our centrality images, the darkest proton sites are the most central, while the lightest are the least central. Neither traps nor long-range paths appear to be highlighted by adjacent flow-IN centrality or adjacent flow-OUT centrality, which take a short-range view of ion flow. To emphasize that adjacent flow-IN centrality does not highlight key path features, Figure 3 also shows the most probable pathway spanning the simulation box for the number of steps with the shortest average estimated time for the path. Based on Tables 1 and 2, 10-step paths starting at *H*_IID_^Far^ are the fastest when there is a fixed proton at the H_I_^Far^ site highlighted in yellow, whereas 8- and 10-step paths are the fastest without a fixed proton. The most probable 8-step path has a shorter individual estimated time than the 10-step path even though both have similar average times and hence, this one is shown in Figure 3b. In both Figure 3a,b, the most probable paths are colored blue, with the limiting path barrier highlighted in red. The limiting barrier for the most probable fastest path in the presence of a H_I_^Far^ proton (yellow) is a rotation with a 0.28 eV barrier, while the limiting barrier for the most probable fastest path without a fixed proton or without vertex removal is a transfer with a 0.24 eV barrier. The limiting barrier move types are consistent with Tables 1 and 2, which show that the 10-step fastest paths in the presence of a fixed proton have limiting barriers of rotation 82% of the time while the 8-step fast paths without the presence of a fixed proton have limiting barriers of intraoctahedral transfer 90% of the time. These values differ from those published in our earlier work,^19^ where calculated vibrational frequencies were used rather than simply the scale of the vibration. The different starting points here reflect our interest in understanding the effect of removing a specific proton site. While there are some high adjacent flow-IN centrality sites on the most probable pathways highlighted in Figure 3, this short-range centrality does not emphasize the paths.

KMC averages which include more diffusion rather than simply looking at the fastest motion in a single direction are shown in the last row of Tables 1 and 2 revealing a greater proportion of intraoctahedral transfers for both scenarios. The most frequent limiting barriers were cataloged, and Figure 3a,b shows the most common limiting barrier for KMC trajectories spanning the simulation box with a green double-headed arrow. The barrier for these steps is 0.42 eV in the presence of a fixed proton and 0.31 eV without a fixed proton. Overall, comparing adjacent flow-IN centrality measures in Figure 3a,b with the most probable long-range pathways and the most often cataloged KMC limiting step reveals little correlation between this most local form of centrality and overall proton conduction.

**Figure 4:**
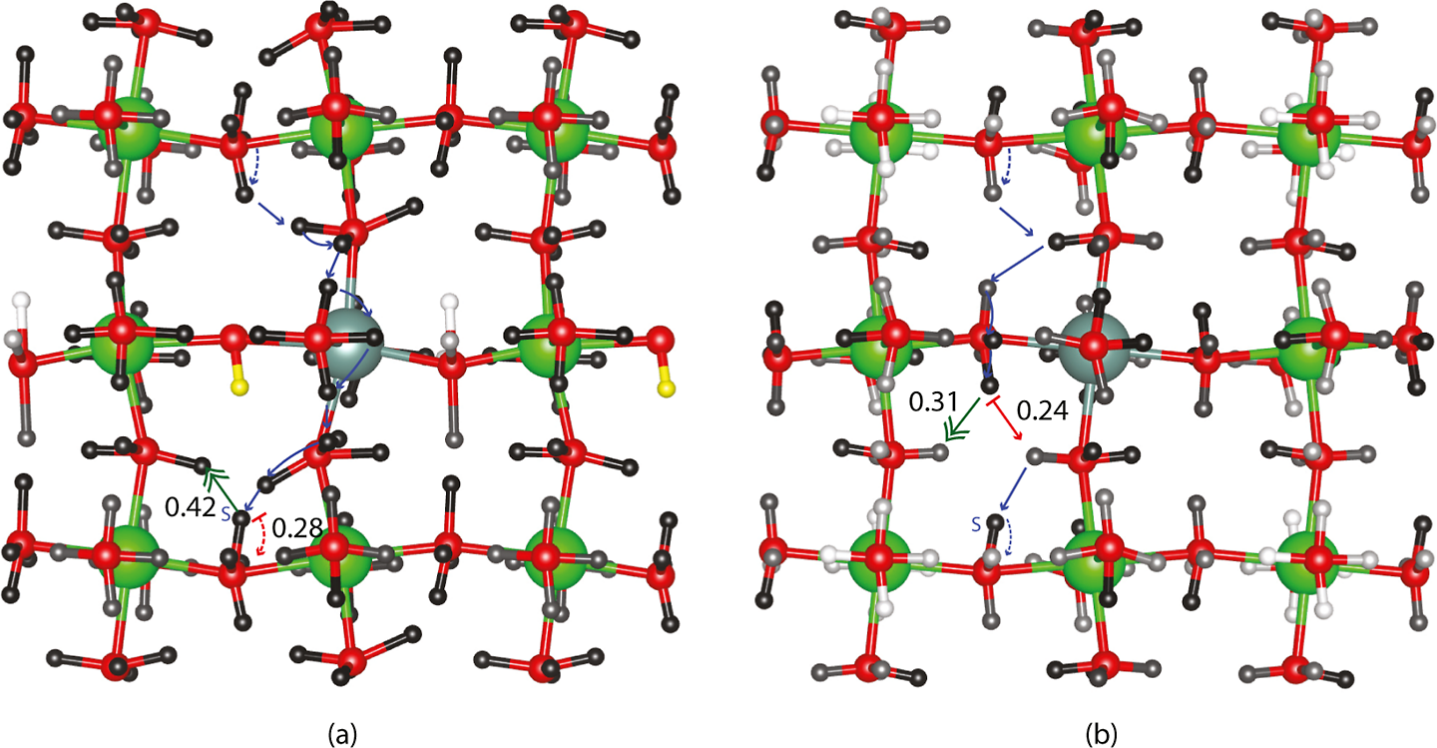
Proton return-flow centrality around a removed vertex (fixed proton) is shown in a dopant plane in (a). Return-flow centrality with all sites available is shown in (b). As in all centrality figures, the most-probable fastest 10-step and 8-step paths are shown with limiting barriers highlighted in red. The KMC most cataloged limiting barrier is emphasized with a double-headed green arrow.

In contrast, return-flow centrality reveals a clear pattern of the most probable paths moving through the most central regions (darkest sites) when all proton sites are accessible, although there is significantly less contrast in centrality with a fixed proton. Figure 4 shows the return-flow centrality for the proton sites in the dopant plane with (a) and without (b) a fixed proton in the site highlighted in yellow, the lowest energy single proton site. Centrality in planes without dopant ions is very low when all sites are accessible, and in fact the most probable paths are on dopant planes when all sites are accessible. However, when the proton is fixed at the yellow highlighted site in Figure 4a, the relative return-flow centrality shown is very similar throughout the doped plane and even in some regions in the undoped planes. Fixing a proton in the location of the lowest energy site removes four different binding sites, the four possible binding locations around an oxygen ion, and changes the centrality picture. Because the lowest energy site in the original graph happens to be the highest return-flow centrality site, removing this vertex and the adjacent ones causes a significant disruption in the graph and hence, a large change in the return-flow centrality image. The magnitude of the unscaled centrality difference before and after removal of the highest centrality site is 93% of the magnitude of the original unscaled centrality. Having an unscaled centrality decrease of nearly twice the original centrality highlights a major network disruption. It is important to note that the images in Figure 4 show scaled centrality measures so that differences in site centrality within each image are visible. This major disruption is highlighted in KMC trajectories, as the average time to cross the simulation box is roughly doubled when a high centrality vertex is removed, as seen by comparing Tables 2 and 1 average estimated times for KMC.

**Figure 5:**
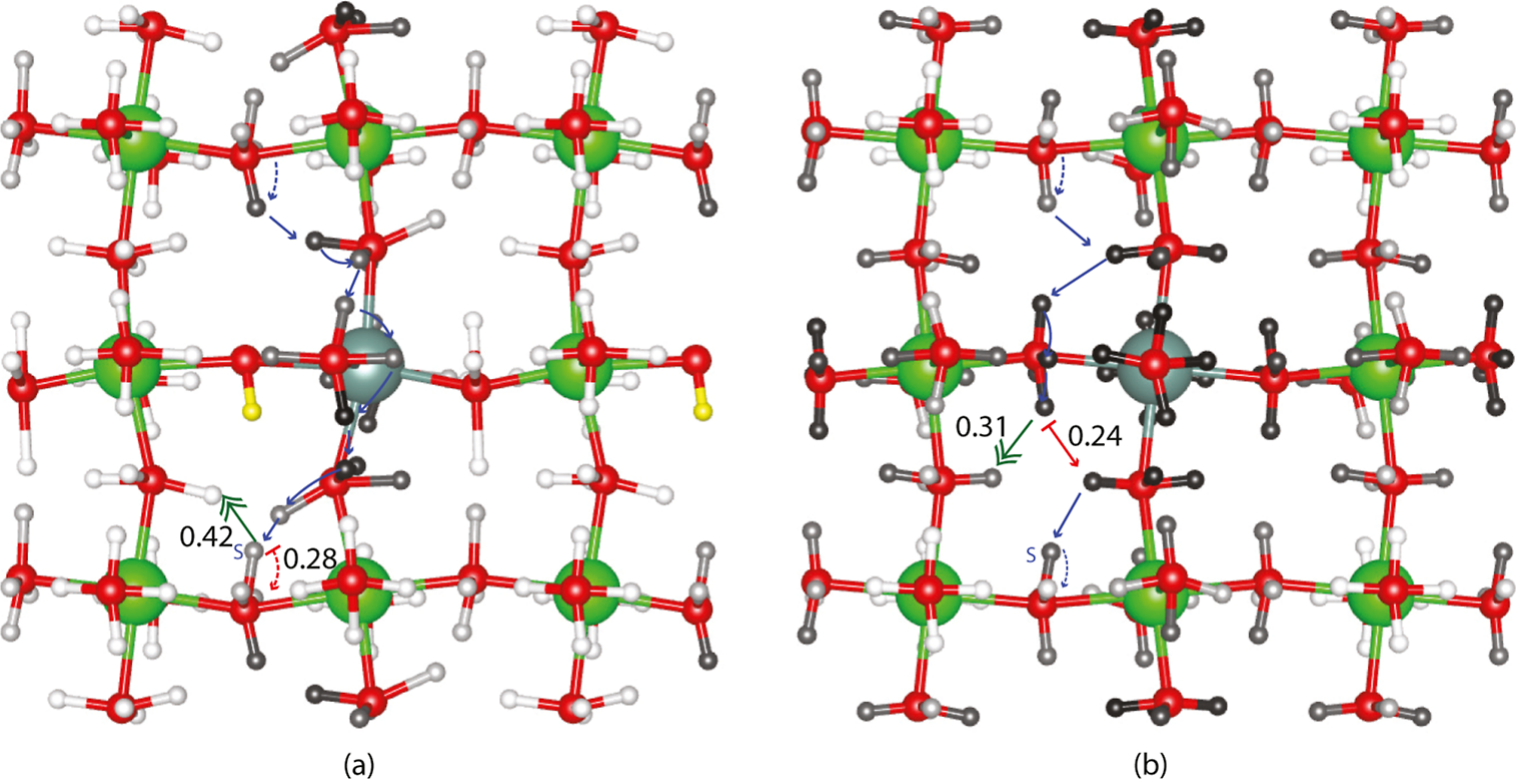
Long-range periodic flow-through centrality around a removed vertex (fixed proton) (a) and with all sites available (b) is shown in a dopant plane. (a) 10-step periodic paths and (b) 8-step periodic paths. As in all centrality figures, the most-probable fastest 10-step and 8-step paths are shown with limiting barriers highlighted in red. The KMC most cataloged limiting barrier is emphasized with a double-headed green arrow.

**Figure 6:**
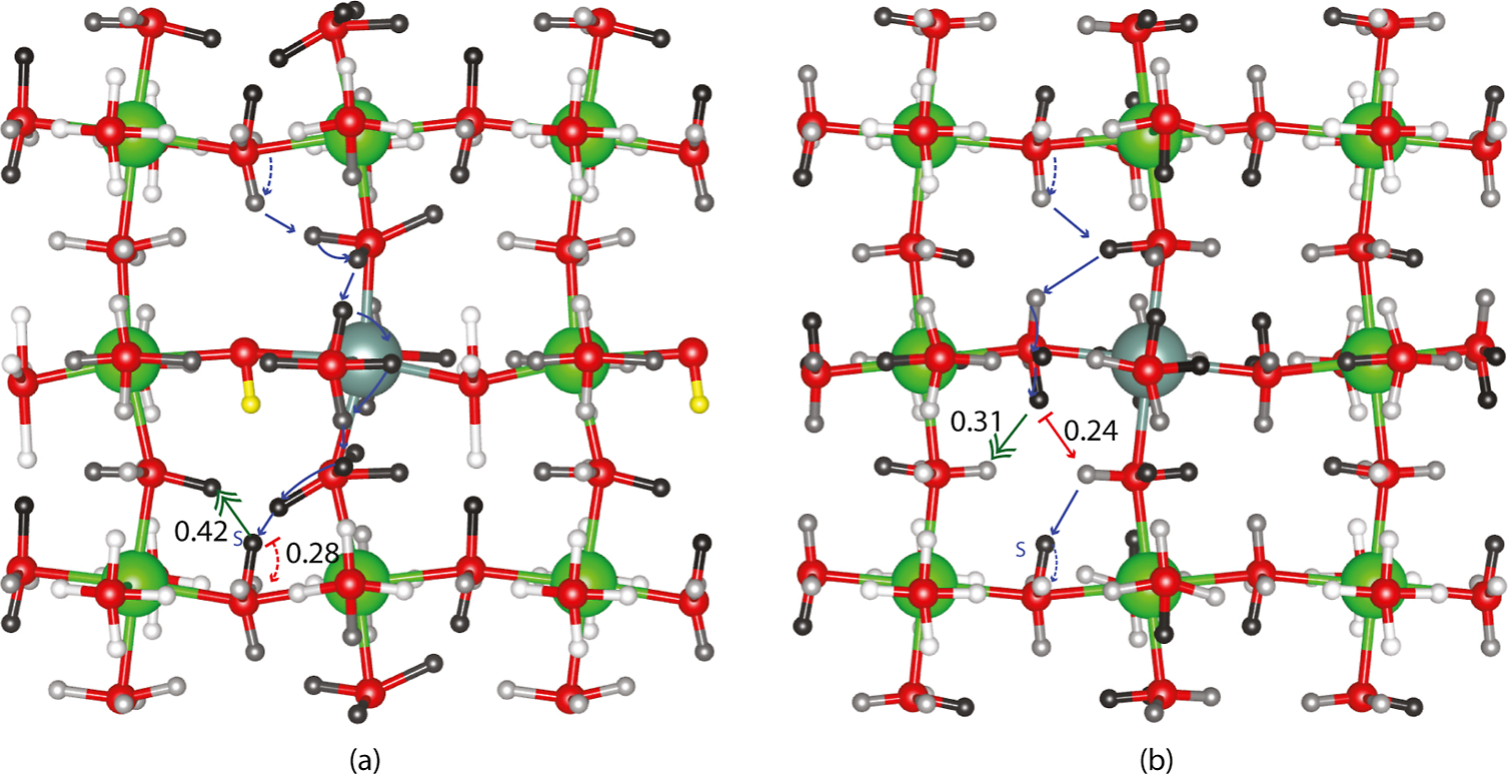
Flow-through centrality for all paths around a removed vertex (fixed proton) (a) and with all sites available (b) is shown in a dopant plane. As in all centrality figures, the most-probable fastest 10-step and 8-step paths are shown with limiting barriers highlighted in red. The KMC most cataloged limiting barrier is emphasized with a double-headed green arrow.

Flow-through centrality shows the most robust correlation with the most probable long-range pathways, and KMC is most often seen limiting barriers to long-range conduction. Figure 5 shows the flow-through centrality with (a) and without (b) the excess proton obtained through averaging over all long-range periodic pathways of 10 and 8 steps, respectively. Notice that the most probable long-range paths are highlighted by the site centrality in addition to the trapped region around the yttrium dopant. Further, the contrast between Figure 5a,b shows how the fixed yellow proton limits the diversity of highly probable long-range path options to be close to the fixed yellow proton as suggested by earlier works.^21,23,24^Figure 6 shows the flow-through centrality with (a) and without (b) the excess proton for all paths. This centrality not only highlights the long-range path regions but also allows the greater meandering through short-range paths available in KMC. In fact, the most often seen KMC limiting barriers to long-range conduction for paths move away from the probable long-range paths. These moves highlighted with the green double-headed arrow go to medium to high flow-through centrality regions when all paths are considered, as seen in Figure 6. Interestingly, while the fixed yellow site is still the most flow-through central node in the original unrestricted graph, the difference in the ratio of the magnitude of the unscaled centrality change vector to the magnitude of the original centrality vector is now just 55%. Instead of the maximum unscaled centrality decreasing by a factor of 10 as occurred in return-flow centrality, the maximum unscaled flow through centrality is of roughly the same magnitude before and after vertex removal, highlighting the greater robustness of flow-through centrality to vertex removal compared to return-flow centrality.

### Robustness of Centrality Measures to Noise

To extend the ways in which time-based centrality measures are used, it is important to be able to calculate the rate constants needed to find them in a greater number of ways. For example, rather than needing to calculate many different binding sites and transition states, it would be convenient to be able to use molecular dynamic trajectories to ascertain which moves are most important and to determine rate constants from the trajectories. Rate constants calculated from molecular dynamics have a significant amount of noise, particularly for rare moves. Centrality found from rate constants with addition of Gaussian noise with mean 0 ps^–1^ and a standard deviation 0.02 ps^–1^ yields nearly the same initial centrality image for both return flow centrality and flow-through centrality. However, using a standard deviation of 0.10 ps^–1^, the centrality pattern is obscured slightly and with a standard deviation of 0.20 ps^–1^, the pattern is completely obscured. At first glance, this bodes poorly for centrality measures calculated from noisy data; however, if small rate constants are approximated using a detailed balance and the larger backward rate constants specifically *k*_*i*,*j*_^small^ = π_*j*_*k*_*j*,*i*_^large^/π_*i*_, restoration of the original centrality image is possible with 0.10 ps^–1^ noise standard deviation and a reasonable but less accurate image can be restored even with a noise standard deviation of 0.20 ps^–1^.

## Conclusions

The three most commonly used centrality measures in network theory have been adapted to consider time rather than the number of steps and renamed to reflect intuition for ion conduction. All have been applied to proton conduction in yttrium-doped barium zirconate when all proton binding sites are available and when one site has been removed to probe proton–proton correlation. Flow-IN centrality highlights sites with the largest flow of ions from the nearest neighbor sites. Such sites give an indication of only fast one-step conduction. Return-flow centrality highlights sites with a fast rate of first returns for the conducting ion. When all vertices or binding sites are accessible, high return-flow centrality sites highlight both conduction traps and highways, as seen in our earlier work.^12,13^ However, when the highest centrality site is blocked, e.g., by another conducting ion, the centrality range can drop significantly, blurring the distinction between sites. Flow-through centrality, which highlights which sites support significant flow of conducting ions, appears more robust to removal of the most central vertices and can be calculated with multiple sets of pathways. When all possible paths are considered, flow-through centrality highlights regions for both traps and highways, highlighting both the most probable long-range pathways and the most probable KMC limiting steps with good contrast, even when the most central vertex is removed. When only long-range periodic pathways are considered, a picture that strongly emphasizes those long-range pathways emerges, but the KMC most probable limiting steps for long-range motion are not highlighted as well. Comparing KMC trajectories to long-range periodic pathways with no internal loops shows that KMC trajectories meander more and those diffusive moves lead to the limiting barrier for long-range motion in the sample system considered. The flow-through centrality including all paths further highlights that when the most central proton site is filled, the highest flow-through centrality sites are nearby. Flow-through centrality results corroborate earlier studies suggesting dual proton motion.^21,23,24^ For example, the proton–proton correlation function calculated from multiproton KMC trajectories showed the highest correlation at distances placing protons in very close proximity suggesting proton pairing.^21^ A systematic exploration of dual proton motion reveals a critical barrier to long-range conduction, which involves a hydroxide rotation near a second proton.^23^ A proton–proton correlation has also been confirmed through X-ray and neutron diffraction experiments.^24^ Conduction ion correlation is important in many systems, especially those with high conduction ion concentrations. Correlated jump analysis highlighted a sodium–sodium motion correlation in sodium-ion conduction.^10^ With its robustness to vertex removal, flow-through centrality adds yet another avenue to probe ion/ion correlation. Finally, centrality measures are robust to noise when a detailed balance is used to calculate the smaller rate constant in a forward/backward pair. Restoration of the centrality image was possible even when introducing random noise with a 0.20 ps^–1^ standard deviation in the rate constants. This may open the way to centrality measures with rate constants calculated by using molecular dynamics.
